# Eight-year follow-up of airway hyperresponsiveness in patients with primary Sjögren’s syndrome

**DOI:** 10.1080/03009734.2016.1239663

**Published:** 2016-11-16

**Authors:** Dora Ludviksdottir, Sigridur Th. Valtysdottir, Hans Hedenström, Roger Hällgren, Björn Gudbjörnsson

**Affiliations:** a Department of Allergy, University Hospital, Reykjavik, Iceland;; b Department of Respiratory Medicine, University Hospital, Reykjavik, Iceland;; c Centre for Rheumatology Research, University Hospital, Reykjavik, Iceland;; d Department of Clinical Physiology, Akademiska Sjukhuset, Uppsala University Hospital, Uppsala, Sweden;; e Department of Medical Sciences, Akademiska Sjukhuset, Uppsala University Hospital, Uppsala, Sweden;; f Faculty of Medicine, University of Iceland, Reykjavik, Iceland

**Keywords:** Airway hyperresponsiveness, follow-up, lung function, Sjögren’s syndrome

## Abstract

**Objective:**

To evaluate in a longitudinal study the influence of airway hyperresponsiveness (AHR) on lung function in patients with primary Sjögren’s syndrome (pSS).

**Methods:**

Lung function was studied over an eight-year period in 15 patients who fulfilled the Copenhagen criteria for primary Sjögren’s syndrome and who were covered in our earlier published study on AHR in patients with Sjögren’s syndrome. Standard spirometry and measurements of lung volumes, diffusing capacity (DLCO), and AHR to methacholine were performed.

**Results:**

A significant decline over time was found in total lung capacity (TLC), vital capacity (VC), forced vital capacity (FVC), functional residual capacity (FRC), and expiratory midflows (FEF_50_). A sign of small airway obstruction (decrease in FEF_50_) at entry correlated with VC at follow-up (*r =* .8, *P <* .003), and the individual change in FEF_50_ during the observation period correlated with the individual change in VC (*r =* .6, *P <* .05). Six patients had increased AHR, and three of them had decreased DLCO. Six of the patients progressively reduced DLCO over time, and five of them had spirometric signs of increased small airway obstruction.

**Conclusions:**

During this eight-year follow-up we observed that one-third of the patients with pSS developed a significant reduction in lung function. Our findings suggest that small airways obstruction and AHR are associated with reduction of VC and development of impaired DLCO as a sign of interstitial lung disease in this group of patients.

## Introduction

Primary Sjögren’s syndrome (pSS) is a chronic autoimmune inflammatory disease that mainly affects exocrine glands of the mucous membranes (1). In addition, a wide spectrum of extraglandular symptoms may occur, e.g. from the respiratory system (2–6). A significant number of patients with pSS have symptoms of xerotracheitis, characterized by a chronic, dry, non-productive cough and dyspnoea. These symptoms have been attributed to dryness in the large airways caused by dysfunction in the tracheal glands due to lymphocyte infiltration (7).

These xerotracheitis symptoms are similar to the symptoms of airway hyperresponsiveness (AHR). In fact, previous studies have demonstrated that the majority of patients with pSS suffer from hyperresponsive airways (8,9). Furthermore, airflow obstruction has been reported in up to 12% of this group of patients (10). It is important to distinguish between bronchial asthma and respiratory symptoms due to pSS, as the Sjögren’s patients may run the risk of developing interstitial pneumonitis and small airway disease, with irreversible lung dysfunction as a possible end result (4).

Few longitudinal studies have been undertaken concerning lung function in patients with pSS (4,11,12), and none of these studies have focused on AHR. Therefore, we have studied whether signs of peripheral or central airflow limitation and AHR are related to changes in lung function over time in this eight-year follow-up study of a group of patients with Sjögren’s syndrome.

## Patients and methods

We have previously reported AHR in a group of patients with pSS (8). Now, eight years later, we have been able to re-evaluate 15 of the 21 previously studied patients. Of the six patients that did not participate at follow-up study, three died, two of myocardial infarction and one of drug intoxication; two were unable to participate for geographical reasons; and one was classified as having systemic lupus erythematosus. Thus, 14 women and 1 man, with a mean age of 60 years (range 46–78) at follow-up, were included in this study. Their disease duration at baseline was 9–15 years (mean 13), with the diagnosis based on the Copenhagen criteria as in the previous study (13). However, all patients who participated at follow-up fulfilled also the classification criteria from 2002 (14).

Four out of 15 patients (26%) had glandular symptoms only, whereas 11 patients (73%) had extraglandular manifestations: Raynaud’s phenomenon (*n =* 9), non-erosive arthritis (*n =* 5), sun sensitivity, pancreatic insufficiency, Waldenström’s macroglobulinemia, and one patient had developed non-Hodgkin’s lymphoma. Eleven patients (73%) had pulmonary symptoms: dry cough (*n =* 7), asthma-like symptoms (*n =* 2), and exertional dyspnoea (*n =* 3). All but one were non-smokers, and none had a history of allergy disorders. Ten patients had a positive ANA, and five had SSA or SSB.

At baseline six patients were treated with glucocorticoids (mean dose: prednisolone 5 mg/day), and three of them were also treated with azathioprine and two with chloroquine. At the follow-up an additional patient was treated with glucocorticoids in combination with chloroquine. None of the subjects was medicated with inhaled corticosteroids.

The study was conducted in accordance with the Declaration of Helsinki and was approved by the Ethics Committee at the Medical Faculty at Uppsala University. Informed consent was obtained prior to the study.

### Lung function measurements

All subjects underwent lung function measurements according to American Thoracic Society (ATS) standards (15), which included measurements of total lung capacity (TLC), functional residual capacity (FRC), and residual volume (RV). Vital capacity (VC), forced vital capacity (FVC), forced expiratory volume in 1 second (FEV_1_), flow volume registrations with maximum expiratory flow (MEF), and flows measured at 50% (FEF_50_) and 25% (FEF_25_) of FVC were measured with a Masterlab body plethysmograph, and diffusion capacity for CO (DLCO) was measured using the Masterlab Transfer test (Eric Jaeger AB, Würtsburg, Germany). Lung function values are presented as a percentage of reference values according to gender, age, and body size (16,17), and values less than 80% of predicted value were considered below normal values.

### Methacholine challenges

The methacholine test was modified from Hargreave’s method (18). A hand-held DeVilbiss 646 nebulizer was used. After an initial test with saline, the patients were tested with double dilutions of methacholine, at 3-min intervals, starting with 1.2 mg/mL up to a maximum dose of 20 mg/mL. The subject inhaled for 2 min, actuating the nebulizer during each inhalation. The nebulizer was weighed before and after each concentration, and the consumed dose was calculated. The inhalation was discontinued when there was a fall in the FEV_1_ of 20% or more below the lowest post-saline value. Test results were expressed as the provocation dose that caused a fall in FEV_1_ of 20% (PD_20_). Degree of AHR was divided into categories based on the PD_20_ value: severe (<0.125 mg methacholine), moderate (0.125–1.29), mild (1.3–5.0), and slight (5.1–9.0).

Thirteen of 15 patients underwent the methacholine challenge test; one had a FEV_1_ of less than 1.0 L/min, and the other was not able to participate in this part of the study. Both these patients demonstrated AHR at baseline.

In addition to PD_20_, we calculated the least-squares slope for methacholine from the regression equation for the percentage decline in FEV_1_ on a cumulative dose of methacholine using all the measured points (18).

### Statistics

The paired *t* test was used to assess changes in lung function and airway responsiveness over time. In comparisons within a group, the Spearman rank correlation test was used. All calculations were done in StatView for Macintosh. A *P* value of <.05 was regarded as statistically significant.

## Results

The individual data on FEF_50_, DLCO, and the response to methacholine (PD_20_) at baseline and at follow-up are presented in Table 1. The results for the whole group illustrate significant decreases in several lung volumes: TLC, VC, FVC, and FRC (Table 2). The patients also had a decrease (*P <* .05) in peripheral airflow (FEF_50_), whereas the measurements of central airway obstruction (MEF) did not change during the study period.

**Table 1.** Results of FEF_50_, DLCO, and methacholine challenge test at baseline and follow-up in 15 patients with primary Sjögren’s syndrome.

	FEF_50_		DLCO		Methacholine PD_20_	
Case no.	Baseline	Follow-up	Baseline	Follow-up	Baseline	Follow-up
1	148	130	75	74	1.80	10
2	57	56	85	97	3.20	9.40
3	36	46	86	92	0.40	0.24
4	114	160	108	80	10	10
5	143	140	129	130	10	10
6	102	109	54	62	10	10
7	120	53	74	64	0.14	0.09
8	74	63	74	76	10	10
9	78	33	68	65	0.76	0.14
10	74	59	74	85	6.60	2.50
11	66	75	82	93	10	10
12	70	60	114	96	10	2.51
13	10	5	53	43	0.62	–
14	99	66	78	83	0.4	–
15	64	52	66	72	0.5	0.15

DLCO = diffusion capacity for CO (% of predicted); FEF_50_ = expiratory flow measured at 50% (% of predicted); PD_20_ = provocation dose which causes a decline in FEV_1_ of 20%.

**Table 2.** Comparison of lung function tests in patients with primary Sjögren’s syndrome, values as a percentage of predicted values according to actual age and body size at baseline and at the eight-year follow-up (mean ± SD).

	Baseline	Follow-up	*P*
TLC	105 ± 20	99 ± 19	<.05
VC	98 ± 26	92 ± 27	<.001
FVC	95 ± 26	89 ± 27	<.05
FRC	121 ± 26	104 ± 23	<.005
FEV_1_	93 ± 30	90 ± 31	ns
FEV_1_/VC	95 ± 17	95 ± 18	ns
FEF_50_	84 ± 38	71 ± 37	<.05
FEF_25_	73 ± 32	68 ± 35	ns
MEF	98 ± 32	99 ± 40	ns
RV	129 ± 40	127 ± 38	ns
DLCO	82 ± 21	81 ± 20	ns
Cst	135 ± 53	120 ± 48	<.05

Cst = static compliance; DLCO = diffusion capacity for CO; FEF_25_ = expiratory flows measured at 25%; FEF_50_ = expiratory flows measured at 50%; FEV% = forced expiratory volume in 1 second as a percentage of VC; FEV_1_ = forced expiratory volume in 1 second; FRC = functional residual capacity; FVC = forced vital capacity; MEF = maximum expiratory flow; TLC = total lung capacity; VC = vital capacity.

**Figure 1.**
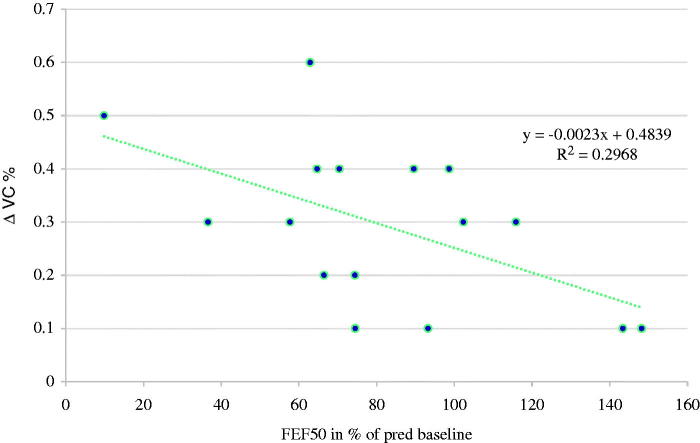
Linear correlation between individual percentage change (Δ) in VC at follow-up and FEF_50_ at baseline in 15 patients with primary Sjögren’s syndrome.

**Figure 2.**
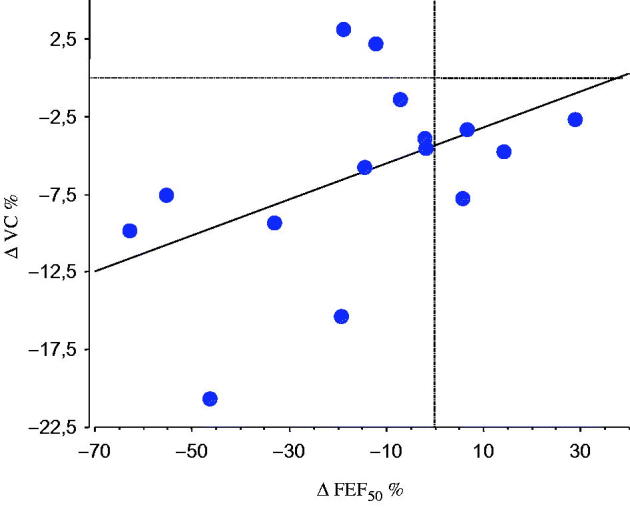
Linear correlation between the individual percentage change (Δ) in FEF_50_ and VC during the observation period in 15 patients with primary Sjögren’s syndrome (*r* = .6, *P <* .05).

When evaluating the airway obstruction over time, we found that FEF_50_ at baseline was correlated with VC at follow-up (*r =* .8, *P <* .003), and there was also an association between the FEF_50_ at baseline and the change (Δ) in VC over time (Figure 1). The individual change in FEF_50_ during the observation period correlated with the individual change in VC (*r =* .6, *P <* .05) (Figure 2).

Seven of 13 (54%) patients with pSS were hyperresponsive to methacholine at baseline, while 6 (46%) were hyperresponsive at follow-up. In the patients with AHR at follow-up, the decrease in DLCO was related to the dose–response slope for methacholine (*r =* .9, *P <* .05). Six (46%) patients had increased AHR over time, and three of them had a reduction in DLCO. Two patients who were AHR-positive to methacholine in the previous evaluation did not demonstrate increased responsiveness to methacholine at follow-up. One of these two patients received no medical treatment, whereas the other started treatment with glucocorticosteroids and hydroxychloroquine during the study period. One patient developed AHR during the study period.

**Figure 3.**
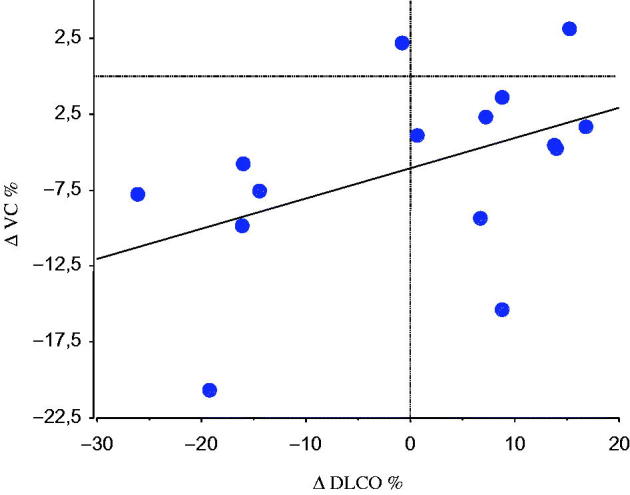
Linear correlation between the individual percentage change (Δ) in DLCO and VC during the observation period in 15 patients with primary Sjögren’s syndrome (*r =* .6, *P <* .05).

The DLCO did not decline in the group during the study period, but three patients who had decreased DLCO at baseline and had AHR progressed in terms of DLCO impairment. Individual changes in DLCO correlated with individual changes in VC (*r =* .6, *P <* .05) (Figure 3). Moreover, we observed that five of six patients whose DLCO decreased progressively during the period also had progression of peripheral airflow limitation. DLCO corrected for alveolar volume did not change, so the decrease in DLCO could not be explained by decreased alveolar volume.

The pulmonary compliance (Cst) was significantly lower at follow-up compared to baseline (*P <* .05).

## Discussion

In the present eight-year follow-up study of patients with primary Sjögren’s syndrome (pSS), signs of increased airflow limitation were found during the observation period. Furthermore, the degree of FEF_50_ at entry and during the observation period was significantly related to the decrease in VC over time. These findings suggest that small airway disease may be an important pathophysiological mechanism in lung involvement in patients with pSS.

The few longitudinal studies which have examined the progression of lung involvement in pSS have shown a minimal deterioration in respiratory function over time (11,12). However, a reduction in the end-expiratory flow in pSS may not always be a permanent sign, but may vary over time. Evidence of obstructive lung disease has been demonstrated by spirometry findings in 20%–50% of patients with pSS (3,10), and HRCT studies have reported bronchiolectasis and bronchiolar and bronchial thickening (19).

AHR is a characteristic finding in bronchial asthma and has been related to bronchial inflammation and oedema of the airway walls (20). Inhaled glucocorticoids decrease AHR in bronchial asthma (21), but seem to have little significant effect on AHR in pSS (22), a finding that further supports the conclusion that there are differences in underlying mechanisms for bronchoconstriction in these diseases. Furthermore, it has been demonstrated using different provocation agents that patients with pSS display different bronchial responsiveness profiles compared to patients with asthma who often show positive response to more than one type of provocation, while patients with pSS more often are only positive to methacholine provocations (23). It is possible that the AHR in pSS may be attributed to an inflammatory process located in the smaller airways. In our study the airway hyperresponsiveness in patients with pSS was associated with a decline in DLCO, a finding which favours the hypothesis that inflammation in the small airways may underlie AHR. The dryness of the bronchial mucosa in pSS may also make the epithelium more vulnerable to damage and probably induces a hyperosmolar stage, thereby predisposing to airway remodelling and AHR. Increased concentration of nitric oxide in the expired air of patients with pSS further provides evidence of a chronic inflammatory process in the airway epithelium in patients with pSS (24).

The clinical significance of decrease in pulmonary compliance in the present study is unknown. However, increased age of the study subjects at follow-up may possibly play a role. In contrast, increased pulmonary compliance has been associated with pulmonary fibrosis, which we did not see in our study group.

Airway disease in pSS appears to be produced both by glandular and non-glandular pathology. The main histological lesion in Sjögren’s syndrome consists of focal lymphocytic infiltrates of exocrine glands (25). This may result in dysfunction of the airway glands, which may give rise to dry and irritating cough – the xerotracheitis symptoms. Extraglandular lymphocytic infiltration of the respiratory system has also been reported in pSS. Endobronchial biopsies have detected follicular lymphocytic bronchiolitis (5,26,27), while bronchoalveolar lavage (BAL) studies reported subclinical alveolitis (28,29), characterized also by lymphocytes. In another study an increased number of alveolar neutrophils were associated with a reduction in DLCO, and abnormal findings on chest HRCTs were found (30). However, our previous study did not show an increased number of inflammatory cells or pro-inflammatory cytokines in the BAL fluid from patients with pSS compared to healthy controls (31). Controversial BAL findings have therefore been reported in pSS. However, several investigators have questioned the meaning of BAL fluid lymphocytosis, as it may not always represent alveolitis but rather reflect bronchial lymphocytic infiltration (7).

Our study has several limitations. Firstly, it is a small study, and although these patients have been reported previously, this group is of interest in context of follow-up. Secondly, results from HRCT would have strengthened our results. Furthermore, our patient group is initially a consecutive series of patients from a specialist clinic. However, we were not able totally to control the care of the patients. We used FEF_50_ as an indicator of small airway obstruction, but FEF_25–75_ might have been more suitable. In spite of these limitations, the result of our study is of interest for those clinicians who are caring for patients with pSS who frequently have pulmonary symptoms.

In summary, the present study further supports that patients with pSS may develop AHR and signs of small airways disease. Spirometry, bronchial challenge tests, and DLCO measurements may be of value to identify patients at risk of developing lung disease associated with Sjögren’s syndrome. Further studies are needed on this issue.
